# Do high risk patients alter their lifestyle to reduce risk of colorectal cancer?

**DOI:** 10.1186/1471-230X-14-22

**Published:** 2014-02-07

**Authors:** Gregory P Tarr, Andrew Crowley, Rhys John, Jonathan B Kok, Ho-Nam L Lee, Hasif Mustafa, Kia M Sii, Rebecca Smith, Sung-Eun Q Son, Lauren J Weaver, Claire Cameron, John D Dockerty, Michael Schultz, Iain A Murray

**Affiliations:** 1 Dunedin School of Medicine, University of Otago, PO Box 913, Dunedin, New Zealand; 2 Preventive and Social Medicine, Dunedin School of Medicine, University of Otago, Dunedin, NZ; 3 Gastroenterology Unit, Dunedin Public Hospital, Dunedin, NZ

**Keywords:** Colorectal cancer, Surveillance, Healthy lifestyle

## Abstract

**Background:**

Colorectal cancer (CRC) may be reduced by healthy lifestyle behaviours. We determined the extent of self-reported lifestyle changes in people at increased risk of CRC, and the association of these reports with anxiety, risk and knowledge-based variables.

**Methods:**

We randomly selected 250 participants who had undergone surveillance colonoscopy for family history of CRC. A telephone interview was conducted, recording demographics and family history. Self-reported lifestyle change due to thoughts about CRC across a range of dietary and lifestyle variables was assessed on a four-point scale. Participants’ perceptions of the following were recorded: risk factor knowledge, personal risk, and worry due to family history. General anxiety was assessed using the GAD-7 scale. Ordinal logistic regression was used to calculate adjusted results.

**Results:**

There were 148 participants (69% response). 79.7% reported at least one healthy change. Change in diet and physical activity were most frequently reported (fiber, 63%; fruit and vegetables, 54%; red meat, 47%; physical activity, 45%), with consumption of tobacco, alcohol, and body weight less likely (tobacco, 25%; alcohol, 26%; weight 31%). People were more likely to report healthy change with lower levels of generalized anxiety, higher worry due to family history, or greater perceived knowledge of CRC risk factors. Risk perception and risk due to family history were not associated with healthy changes.

**Conclusions:**

Self-reported lifestyle changes due to thoughts about CRC were common. Lower general anxiety levels, worries due to family history, and perceived knowledge of risk factors may stimulate healthy changes.

## Background

Colorectal cancer (CRC) is a major cause of morbidity and mortality, and this burden may be reduced by the adoption of a range of healthy behaviours, including screening/surveillance, and a choice of lifestyle factors [1].

Changing patterns of screening behaviour and healthy lifestyle factors have been linked to decreased incidence of CRC. Since 1975, CRC incidence has fallen by around 30% in the United States. This reduction appears to be equally attributable to increased screening/surveillance, and to adopting healthy lifestyle behaviours [2].

Cancer risk factors are often reported in popular media, and it appears that public knowledge of CRC risk factors [3,4] parallels those for cardiovascular disease [5,6]. In a large Dutch cohort, increasing prevalence of healthy lifestyle behaviours was associated with a reduction in incidence of CRC [7]. At the population level, moderate variation in levels of exercise, tobacco smoking, fruit/vegetables/fiber and alcohol intake appeared to explain around one quarter of the incidence of CRC [7].

Current strategies for modifying healthy lifestyles have had some success in improving risk profiles [8]. People undergoing CRC screening appear to spontaneously adopt healthy lifestyle behaviours for at least a short time around the time of screening [9], suggesting that concerns about CRC may provide a stimulus for positive choices, and people are adherent to screening recommendations are also more likely to adhere to other healthy behaviours [10]. A substantial body of research has linked various factors with the uptake of screening. Risk perception [11] and knowledge [12] both influence the choice to be screened, and multiple studies have linked general anxiety symptoms to increased screening uptake [13-16], although this has been disputed [17-19]. Cancer-specific worry may be a better predictor of screening [20].

While screening and surveillance are important factors in reducing the burden of CRC, lifestyle based harm reduction strategies will likely also play an important part [21]. In light of these findings, it is important to know what promotes people to adopt healthy lifestyles, especially in high risk groups such as familial CRC. However, literature on this is sparse. Qualitative studies highlight the current mismatch in public understanding of CRC, with patients undergoing CRC screening perceiving adenomas as a minor problem [22], having lack of understanding of CRC risk factors [23] and some being skeptical about the need for lifestyle change [22,23]. On a more positive note, some patients were amenable to changing their lifestyles in order to decrease risk of CRC [22,23].

The aims of our study were to describe the extent of self-reported changes in healthy lifestyle behaviours in a patient group at increased risk of CRC, and the effect risk perception, anxiety and family history of CRC had on the adoption of these lifestyles.

## Methods

### Study population

Patients who underwent colonoscopy surveillance between 1996 and 2012 at Dunedin Hospital, New Zealand for a family history of CRC were eligible for inclusion. Participants were excluded if they were aged over 75 years at recruitment (the age at which surveillance colonoscopy is discontinued, in keeping with national guidelines), had a personal history of CRC, symptoms of CRC or inflammatory bowel disease. Participants were randomly selected from a clinical endoscopy database of 1,086 patients.

### Study questionnaire

Potential participants were provided with an information sheet and visual aid by mail, then telephoned to obtain verbal consent. The survey was administered using a labeled visual scale to assist in risk estimation. The telephone interview was standardized across ten interviewers. The methods are reported previously elsewhere [24]. Briefly, demographic and clinical details were collected during the interview, and additional clinical details were obtained from hospital records.

Demographic information is shown in Table 1. Participants reported family history of cancer for first- and second-degree relatives, including type of cancer and age at diagnosis, confirmed by review of hospital records. The number and year of surveillance colonoscopies were recorded from hospital records including colonoscopy outcomes. NZDep2006 is a measure of socioeconomic deprivation derived from census indices, based on the street address of each participant [25].

**Table 1:** Demographic and clinical characteristics of the study population

	**N = 148**
Age, years	57.9 (56.5 to 59.3)
Sex, male	56 (37.8%)
NZ European	142 (96.0%)
NZDep2006 †	5.3 (4.9 to 5.7)
Alcohol, median (IQR) ‡	3 (1 – 7)
No alcohol use	36 (24.3%)
Tobacco use	
Never	68 (46.3%)
Ex	66 (44.9%)
Current	13 (8.8%)
Marital status	
Married/de facto	109 (73.6%)
Single/widowed	22 (14.9%)
Divorced/separated	17 (11.5%)
Education completed	
Primary	6 (4.1%)
Secondary	75 (50.7%)
Tertiary	66 (44.6%)
Personal history of non-CRC cancer §	23 (15.5%)
Colonoscopies, median (IQR)	1 (1 – 3), range 1 – 7
Polyps removed, median (IQR)	1 (0 – 2), range 0 – 120
First degree relatives, median (IQR)	1 (1 – 2)
Age of first degree relative at diagnosis	49.5 (47.5 to 51.6)
Second degree relatives, median (IQR)	1 (0 – 2)
Total relatives with any cancer, median (IQR) ††	3 (2 – 4)
NZGG category 1 (~12% lifetime risk)	15 (10.1%)
NZGG category 2 (~18 to 36% lifetime risk),	66 (44.6%)
NZGG category 3 (up to 50% or greater lifetime risk)	57 (38.5%)
Second degree relatives only (risk approximately 5.9%)	10 (6.8%)

Results are mean (95% confidence interval or number (percentage), unless stated otherwise.

† New Zealand Deprivation Index 2006.

‡ Units of alcohol consumed in one average week.

§ Participants with a history of CRC were excluded.

Number of relatives with CRC.

†† Number of relatives (first- or second-degree) with any cancer.

CRC = colorectal cancer; IQR = interquartile range; NZGG = New Zealand Guidelines Group.

Each participant was asked for their perception to what degree CRC is genetic, how knowledgeable they were about CRC lifestyle risk factors, and to what degree they had altered the following lifestyle risk factors (reducing weight, red/processed meat, alcohol and smoking; increasing dietary fiber, fruit/veges, and physical activity) because of concerns about CRC. Participants were prompted with regard to each lifestyle factor (i.e. “how much have you reduced tobacco/cigarette consumption because of thoughts about CRC”). We used a four point, ordinal scale (Not at all, A little, Somewhat, Very much so). The questions regarding behavioural change enquired about subjective, self-perceived change since the initiation of surveillance. There was no formal validation of patient responses to measured behaviours. For patients who did not smoke/consume alcohol, their change in behaviour was labelled as “Not applicable” We asked participants to estimate their personal lifetime risk of CRC without colonoscopy surveillance on a 0 – 100% scale. The Generalized Anxiety Disorder 7 scale (GAD-7) was administered as per authors recommendations [26].

The Lower South Regional Ethics Committee approved the study.

### Study measures

Risk of CRC was estimated by New Zealand Guidelines Group (NZGG) recommendations [27]. These guidelines classify risk by the presence of family history and features of genetic CRC syndromes. Briefly, NZGG categorizes patients into four risk categories on the basis of the number of first degree relatives with histories of CRC and their age at first diagnosis [27]. NZGG category was determined by a team of three investigators (GPT, AC, JR) and from clinical documentation, and agreement between the two was good (Kappa 0.76).

### Statistical analysis

Statistical analysis was performed using Stata 10.0 (StataCorp) and StatView version 5.01 (SAS Institute). Results are shown as mean (95% confidence interval) or number (percentage) unless otherwise stated. Continuous variables were compared between categories using ANOVA, and discrete variables were compared using the Chi-square test. To assess the relationship between anxiety, worry due to family history, and perceived knowledge and behavioural change, ordinal logistic regression including adjustments for age, sex and socioeconomic status was performed. A P value of < 0.05 was considered statistically significant. This was an exploratory study, and there was no previous data to base a sample size calculation on. Pearsons correlation was used to measure the association between different the number of healthy lifestyles adopted, and anxiety, worry and knowledgeability.

The primary outcome was the proportion of participants reporting changes in each healthy behaviour. Secondary outcomes included whether risk perception, generalized anxiety, worry due to family history and risk due to family history were associated with changes in a composite of all recorded healthy behaviours. To adjust these analyses for confounding factors, ordinal logistic regression was used. Odds ratios compared either a one-point increase, or SD increase of exposure variable with the number of healthy lifestyle behaviours changed “Somewhat” or “Very much so” compared to those changed “Not at all” or “A little”.

## Results

### Recruitment and demographics

Full details of the study population including demographics are reported elsewhere [24]. Of 250 people initially sampled, 34 were ineligible, 47 could not be contacted and 21 declined consent. The exclusions were as follows: previous CRC n = 11, inflammatory bowel disease n = 4, not in surveillance program = 1, duplicate n = 1, deceased n = 4, aged over 75 years n = 13. The remaining 148 (69%) participated. Those excluded were younger than respondents (55.3 years old [95% CI 53.0 to 57.5] vs. 57.9 [95% CI 56.5 to 59.3]; p < 0.05), but similar by sex (42.4% vs. 37.8% male; P = 0.63). The clinical and demographic details including NZGG-based risk groups of the included population are given in Table 1. The majority were middle aged (range 35 to 74 years), female, married or in a de-facto relationship, had undergoing a single colonoscopy and were at least three times the population risk of developing colorectal cancer in their lifetimes.

### Self-reported knowledge of colorectal cancer risk factors and behavioural change

The majority of participants (77.7%) believed that CRC risk was genetic and relatively uninfluenced by lifestyle behaviours (Table 2). Two-thirds of patients worried “Sometimes”, “Often” or “Always” about their risk based on their family history. The majority felt they were “Somewhat” or “Very much so” aware of lifestyle risk factors for CRC.

**Table 2:** GAD-7 score, anxiety about surveillance, and worry about family history of colorectal cancer

	**N = 148**
To what extent is CRC genetic and will happen regardless of how we live?	
Not at all	4 (2.7%)
A little	29 (19.6%)
Somewhat	62 (41.9%)
Very much so	53 (35.8%)
Do you worry about developing CRC based on your family history?	
Not at all	18 (12.2%)
A little	36 (24.3%)
Sometimes	62 (41.9%)
Often	25 (16.9%)
Always	7 (4.7%)
I am aware of lifestyle risk factors for CRC?	
Not at all	6 (4.1%)
A little	27 (18.2%)
Somewhat	52 (35.1%)
Very much so	63 (42.6%)
Global GAD-7 score	4.4 (3.7 to 5.1)
Nervous/anxious	0.8 (0.6 to 1.0)
Unable to stop/control worrying	0.5 (0.4 to 0.7)
Worrying too much	0.7 (0.6 to 0.9)
Trouble relaxing	0.7 (0.5 to 0.9)
Restless	0.4 (0.3 to 0.5)
Irritable/annoyed	0.7 (0.6 to 0.8)
Afraid that something awful/bad might happen	0.4 (0.3 to 0.5)

Results are mean (95% confidence interval) or number (percentage).

CRC = colorectal cancer; GAD-7 = Generalized Anxiety Disorder scale – 7.

**Figure 1:**
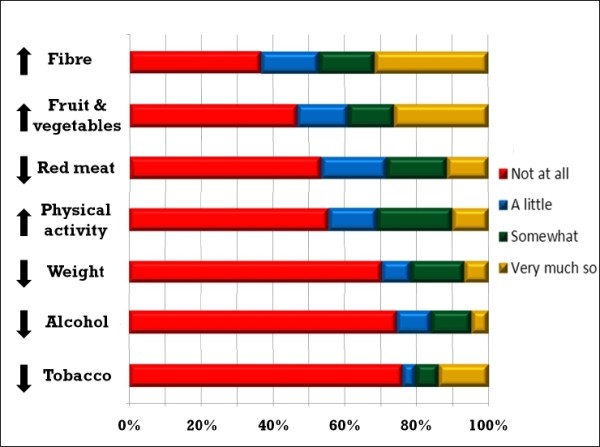
**Self-reported change in healthy behaviours due to thoughts about colorectal cancer risk.** Proportions of patients reporting each degree of adoption of specific healthy behaviours due to thoughts about colorectal cancer. Behaviours are ranked in descending order of likelihood of reported change.

Most (79.7%, n = 118) reported altering at least one lifestyle variable to reduce their CRC risk (Figure 1). For most, this meant dietary change. Thirty participants (20.2%) reported no changes. The median number of healthy behaviours adopted was two. A small number of participants (4.1%, n = 6) reported adopting all healthy behaviours at least somewhat. Relatively few reduced their weight, alcohol intake or tobacco use due to concern over CRC risk.

The reporting of changes in dietary changes were correlated with each other (reducing red meat/increasing fiber, fruit and vegetables (r = 0.36 to 0.5) Reporting reducing weight was correlated with decreasing red meat (r = 0.55) and alcohol (r = 0.2), increasing fiber (r = 0.38), physical activity (r = 0.40) and fruit/vegetables (r = 0.48).

Increased physical activity was correlated with reducing alcohol (r = 0.19) and weight (r = 0.40), increasing fruit and vegetables (r = 0.55) and physical activity (r = 0.55). Decreased alcohol intake was correlated with increasing fruit and vegetable intake (r = 0.25) and decreased weight (r = 0.20).

No other variables were correlated with reported changes in tobacco consumption.

### Influence of knowledge, risk perception and anxiety on reported behaviour change

Global GAD-7 score and sub-scale items are shown in Table 2. The average anxiety levels in our study population correlate with ‘Mild to Moderate’ anxiety [26]. The influences of perceived knowledge, risk perception and anxiety on reported behaviour change after adjustment for age, sex and socioeconomic status are shown in Table 3.

**Table 3:** Adjusted odds ratios for the association of knowledge, anxiety and worry with adoption of healthy behaviours

	**Adjusted odds ratio†**	**95% confidence interval**	**P value**
Perceived knowledge of CRC risk factors			
None	–	–	–
A little	4.16	0.06 to 25.04	0.12
Somewhat	5.01	0.91 to 27.61	0.64
Very much so	9.27	1.69 to 50.72	0.01
Are genes the main cause of CRC?			
Not at all	–	–	–
A little	2.58	0.40 to 16.65	0.32
Somewhat	2.71	0.44 to 16.65	0.28
Very much so	3.79	0.61 to 23.68	0.15
Perceived risk of CRC, per SD increase	1.30	0.97 to 1.75	0.083
Global GAD-7 score, per SD increase	0.66	0.49 to 0.89	0.007
Worry about CRC due to family history			
Never	–	–	–
A little	3.34	1.11 to 10.17	0.033
Sometimes	2.96	1.05 to 8.33	0.040
Often/always‡	4.71	1.55 to 14.37	0.006

† Adjusted for age, sex, and NZDep score.

‡ Often/always combined due to low numbers reporting “always”.

Odds ratios are for the number of behaviours participants rated they had changed “Somewhat” or “Very much so” compared to “Not at all” or “A little”. CRC = colorectal cancer; GAD-7 = Generalized Anxiety Disorder scale – 7.

Perceived knowledge of CRC risk factors and specific worry about CRC due to family history were positively associated with reported healthy behaviour change. Higher generalized anxiety symptoms were associated with a lower number of healthy behavioural changes.

The odds of adopting at least one behaviour “Somewhat or Very much so” were 4.71 for those who worried about CRC due to family history “Often or Always”, and 9.27 for those who perceived they were “Very much so” knowledgeable about CRC risk factors. The odds of adopting each behaviour “Somewhat or Very much so” were 0.66 for each SD increase in GAD-7 score.

Worry due to family history was modestly correlated with agreeing that CRC was due to genetics (r = 0.33) and perceived knowledgeability about CRC risk (r = 0.21). Thoughts about the role of genetics and perceived knowledgeability not correlated.

There was no association with either NZGG-based risk, or the concordance of perceived risk and NZGG-based risk, and behaviour change. There was no association of reported adoption of healthy behaviours with CRC risk perception or whether participants believed genetics to be the primary cause of CRC.

Both GAD-7 and worry due to family history remained significantly associated with reported behavioural change when both variables were included in the model, indicating they are independent predictors of reported behavioural change.

The association between either GAD-7 or worry due to family history and healthy behaviours was not diminished when perceived knowledge of risk factors was included in a multivariate model, suggesting the mechanism by which anxiety relates to healthy behaviours is not mediated through increased knowledge of risk factors.

## Discussion

Participants believed that CRC was mainly influenced by genetics, but self-reported healthy change due to concerns about CRC was still common. Participants with lower anxiety levels, higher worry due to family history of CRC, and greater perceived knowledge of CRC risk factors were more likely to report change in lifestyle behaviours to reduce risk. Participants were more likely to report changing dietary variables and exercise, than reducing body weight, alcohol intake and tobacco smoking.

Most of our high-risk population reported adopting at least one healthy behaviour they attributed to thoughts about colorectal cancer. Patients may adopt healthy behaviours around the time of CRC screening [9], although this has been disputed [28,29]. The benefits of interventions to produce behaviour change is small [8] compared to the normal variation of these factors amongst the general population [7]. Our findings suggest that thoughts about the risk of CRC may stimulate many patients to adopt some healthy behaviours, the exposure to the stresses associated with familial cancer and colonoscopy surveillance may represent a “teachable moment” allowing increased uptake of healthy behaviours.. Furthermore, patients may overestimate the magnitude of their lifestyle change, and further support from medical professionals may increase the likelihood that the changes made are clinically significant.

There are at least two explanations why CRC risk factors may be changed in the reported frequencies. The first is that dietary variables and physical exercise are among the most widely known risk factors in the general population [4]. Studies have found that knowledge of dietary risk factors for CRC including red meat [3,4], fruit and vegetables [4], and lack of dietary fiber [3], was good in the general population, with a smaller proportion of people knowing about smoking [3,4], and lack of physical exercise [3,4] as contributing factors.

Secondly, it may be easier to change diet than to alter smoking, alcohol intake or body weight.

We investigated factors associated with self-reported change due to concerns about CRC. Perceived risk of CRC and true risk due to family history were not associated with healthy behaviours. Greater levels of general anxiety, specific worry about CRC due to family history and perceived knowledge about risk factors were all associated with an increased likelihood of reporting healthy behaviour adoption.

Most of our study participants had low to moderate levels of general anxiety, with few with high anxiety scores. This association between moderate levels of anxiety and adoption of healthy behaviours is consistent with previous research with breast cancer [15,30]. In contrast, high levels of general anxiety have been linked to maladaptive behaviour patterns [19]. However it appears that the relationship between specific worry and behavioural change is stronger and more consistent than that of general anxiety and behavioural change [13,20,30]. Our findings suggest that both lower general anxiety and higher specific worry were independently associated with the likelihood of reporting behavioural change. There have been limited studies on the relationship between general anxiety and self-reported behavioural change, but Bowen et al. [15] found that women with moderate levels of anxiety were more likely to report that they ate a low fat diet.

We found no association between perceived risk of CRC and behavioural change. Other studies have linked elevated risk perception to the uptake of cancer screening [11]. Participants were asked specifically to estimate their risk had they not undergone surveillance. Thus the lack of association between risk perception and reported behavioural change may be due to participants re-appraising their risk after undergoing colonoscopy, and this risk estimate not being currently relevant to their decision-making.

Those with higher perceived risk were likely to attribute a genetic cause to CRC. This might reflect a degree of fatalism amongst those with elevated risk perception, which may have played a role in inhibiting lifestyle behavioural change [31].

Greater perceived knowledge being associated with a greater likelihood of colonoscopy screening uptake with both knowledge about the need for colonoscopy screening [32], and general knowledge about CRC [33] both being important modifying factors. We are not aware that it has been reported as influencing healthy lifestyle changes however.

### Limitations

We prompted participants by enquiring whether they had adopted specific healthy behaviours, which may have led to over-reporting of healthy choices. Furthermore, a number of our questions were about subjective perception. While this provides insight into patients’ thoughts, it also decreases the external validity of the findings.

This was a cross sectional study. Future studies should ideally assess the components of risk perception, anxiety and behavioural change longitudinally before, during and after participation in a surveillance program. Our study focused on patients undergoing surveillance, future studies could compare adoption of health behaviours with low risk groups.

Our study population was predominantly NZ European, potentially limiting generalization to people of other cultures.

There is no model for assessing the risk of CRC that includes measures of lifestyle risk factors that has been validated in our population or is in regular use. Calculated risk due to family history as assessed by NZGG category may not completely reflect the true risk for each individual although is the tool used in clinical practice.

Participant recall has been shown to underestimate the prevalence of CRC in the family history [34]. We validated the family history from the clinical records of each participant, but cannot be certain that we have not under-estimated their true CRC risk attributable to family history.

We had a relatively good response rate, although those excluded were slightly but significantly younger than those included, raising the possibility of selection bias although it would appear unlikely to have influenced our conclusions.

### Implications for future practice

Clinicians should recognize that behavioural risk factor awareness and reported attempts to change are widespread in this high-risk population and support may be indicated. Further research is required to establish risk markers for patients who are less likely to improve lifestyle factors.

## Conclusion

Our study found that participants undergoing colonoscopy surveillance for a family history of CRC perceive themselves to be knowledgeable about lifestyle risk factors for CRC and frequently report attempts to change healthy lifestyle risk factors. Lower general anxiety, higher specific worry due to family history, and perceived knowledge about CRC risk factors were independently associated with self-reported behavioural change.

## Abbreviations

CRC: Colorectal cancer; NZGG: New Zealand Guidelines Group; GAD-7: Generalised Anxiety Disorder scale – 7; ANOVA: Analysis of variance; IQR: Interquartile range; NZDep: New Zealand Deprivation Index 2006; SD: Standard deviation.

## Competing interests

The authors declare that they have no competing interests.

## Authors’ contributions

All authors were involved in the design of the study. GPT, AC, JR, JBK, HLL, HM, KMS, RS, SES and LJW conducted the data collection. Statistical analysis was conducted by GPT and CC, and all authors were involved in the interpretation. The manuscript was prepared by GPT, AC, RJ, RS, CC, JDD, MS and IAM. All authors read and approved the final manuscript.

## Pre-publication history

The pre-publication history for this paper can be accessed here:

http://www.biomedcentral.com/1471-230X/14/22/prepub
